# Age-Dependent Presentation and Clinical Course of 1465 Patients Aged 0 to Less than 18 Years with Ovarian or Testicular Germ Cell Tumors; Data of the MAKEI 96 Protocol Revisited in the Light of Prenatal Germ Cell Biology

**DOI:** 10.3390/cancers12030611

**Published:** 2020-03-06

**Authors:** Gabriele Calaminus, Dominik T. Schneider, Dietrich von Schweinitz, Heribert Jürgens, Nacera Infed, Stefan Schönberger, Thomas A. Olson, Peter Albers, Christian Vokuhl, Raimund Stein, Leendert Looijenga, Jalid Sehouli, Martin Metzelder, Alexander Claviez, Michael Dworzak, Angelika Eggert, Birgit Fröhlich, Nicolas U. Gerber, Christian P. Kratz, Jörg Faber, Thomas Klingebiel, Dieter Harms, Ulrich Göbel

**Affiliations:** 1 Department of Pediatric Hematology and Oncology, University Hospital Bonn, 53113 Bonn, Germany; Stefan.Schoenberger@ukbonn.de; 2 Clinic of Pediatrics, Municipal Hospital, 44137 Dortmund, Germany; Dominik.Schneider@klinikumdo.de; 3 Department of Pediatric Surgery, University of Munich, 80337 Munich, Germany; Dietrich.Schweinitz@med.uni-muenchen.de; 4 Pediatric Hematology and Oncology, University Children’s Hospital Muenster, 48149 Münster, Germany; jurgh@ukmuenster.de (H.J.); birgit.froehlich@ukmuenster.de (B.F.); 5 Coordination Center for Clinical Studies, University Düsseldorf, 40225 Düsseldorf, Germany; nacerainfed@aol.com; 6 Aflac Cancer and Blood Disorders Center, Children’s Healthcare of Atlanta, Emory University, Atlanta, GA 30322, USA; Thomas.Olson@choa.org; 7 Department of Urology, University Hospital Düsseldorf, Medical Faculty Heinrich-Heine University, 40225 Düsseldorf, Germany; Peter.Albers@med.uni-duesseldorf.de; 8 Section of Pediatric Pathology, Department of Pathology, Universitiy Hospital Bonn, 53127 Bonn, Germany; christian.vokuhl@ukbonn.de; 9 Department of Pediatric, University Medical Centre Mannheim, Adolescent and Reconstructive Urology, 68167 Mannheim, Germany; raimund.stein@umm.de; 10 Princess Máxima Center for Pediatric Oncology, 3584 Utrecht, The Netherlands; l.looijenga@prinsesmaximacentrum.nl; 11 Department Gynecology and Center of Gynecological Oncology, Charité University Medicine Berlin, 13353 Berlin, Germany; Jalid.Sehouli@charite.de; 12 Department of Pediatric Surgery, Medical University Vienna, 1090 Vienna, Austria; martin.metzelder@meduniwien.ac.at; 13 Campus Kiel, Department of Pediatric Oncology, Medical University of Schleswig-Holstein, 24105 Kiel, Germany; Alexander.Claviez@uksh.de; 14 St. Anna Children’s Hospital and Children’s Cancer Research Institute, Medical University Vienna, Pediatric Clinic, 1090 Vienna, Austria; michael.dworzak@stanna.at; 15 Department of Pediatric Oncology and Hematology, Charité University Medicine Berlin, 13353 Berlin, Germany; angelika.eggert@charite.de; 16 Department of Oncology, University Children’s Hospital, 8032 Zürich, Switzerland; nicolas.gerber@kispi.uzh.ch; 17 Hannover Medical School, Pediatric Hematology and Oncology, 30625 Hannover, Germany; kratz.christian@mh-hannover.de; 18 Department of Pediatric Hematology/Oncology/Hemostaseology, University Medical Center of the Johannes Gutenberg University Mainz, Center for Pediatric and Adolescent Medicine, 55131 Mainz, Germany; joerg.faber@uni-mainz.de; 19 Department of Pediatric Hematology/Oncology/Hemostaseology, Goethe-University Frankfurt, University Hospital for Children and Adolescents, 60590 Frankfurt am Main, Germany; thomas.klingebiel@kgu.de; 20 Department of Pathology, University of Kiel, 24105 Kiel, Germany; d.harms@online.de; 21 ESPED Surveillance Unit, University Düsseldorf, Coordination Center for Clinical Studies, 40225 Düsseldorf, Germany; goebelu@arcor.de

**Keywords:** germ cell tumors, ovary, testis, children and adolescents, age, sex, histology

## Abstract

Objective: To evaluate prognostic factors in pediatric patients with gonadal germ cell tumors (GCT). Methods: Patients <18 years with ovarian and testicular GCT (respectively OGCT and TGCT) were prospectively registered according to the guidelines of MAKEI 96. After resection of the primary tumor, patients staged ≥II received risk-stratified cisplatin-based combination chemotherapy. Patients were analyzed in respect to age (six age groups divided into 3-year intervals), histology, stage, and therapy. The primary end point was overall survival. Results: Between January 1996 and March 2016, the following patients were registered: 1047 OGCT, of those, 630 had ovarian teratoma (OTER) and 417 had malignant OGCT (MOGCT); and 418 TGCT, of those, 106 had testicular teratoma (TTER) and 312 had malignant TGCT (MTGCT). Only in MTGCT, older age correlated with a higher proportion of advanced tumors. All 736 teratomas and 240/415 stage I malignant gonadal GCT underwent surgery and close observation alone. In case of watchful waiting, the progression rate of OGCT was higher than that of TGCT. However, death from disease was reported in 8/417 (1.9%) MOGCT and 8/312 (2.6%) MTGCT irrespective of adjuvant chemotherapy and repeated surgery. Conclusions: The different pathogenesis and histogenesis of gonadal GCT reflects sex- and age-specific patterns that define clinically relevant risk groups. Therefore, gender and age should be considered in further research on the biology and clinical practice of pediatric gonadal GCT.

## 1. Introduction

Teratomas (TER) and malignant germ cell tumors (MGCT) constitute a heterogeneous group of germ cell tumors (GCT). Malignant testicular gonadal germ cell tumors (MTGCT) are the most frequent solid cancer of men aged 18 to 45 years and encompass about 92.5% of all MGCT. In comparison, malignant ovarian GCT (MOGCT) and malignant extragonadal GCT (MEGCT) are rare and account for only 3.9% and 3.6% of all MGCT, respectively [1,2,3]. Of note, about 40% of pediatric MGCT develop at extragonadal sites, demonstrating the difference in clinical presentation of the various entities; among gonadal GCT in children and adolescents, 60% are MOGCT and 40% are MTGCT. Detailed analysis of epidemiological GCT data has demonstrated characteristic patterns and a significant correlation between gender and age in respect to tumor histology, site, and stage [4]. The prognosis of GCT correlates with therapy, in particular with the completeness of tumor resection [5,6,7]. In MGCT, prognosis has dramatically improved with the introduction of cisplatin-based chemotherapy combinations in the 1970s [8]. Of note, the prognostic International Germ Cell Classification Consensus (IGCCC) for adult males with metastatic MGCT can only be used in part to stratify pediatric GCT patients, as approved prognostic factors are different [9,10].

As postulated by Teilum more than 50 years ago, TER and MGCT develop from transformed primordial germ cells (PGC) [11]. Molecular genetic analyses of pediatric MGCT revealed distinct biological differences between childhood MGCT compared to those in adolescents and adults [12,13]. These studies were instructive to classify GCT in children and adolescents into two main groups: type I-GCT of childhood resembling TER or yolk sac tumor (YST) histology with retained biparental or partially erased genomic imprinting and frequent loss of chromosome 1p and 6q; type II-GCT with often mixed GCT histology presenting a complete loss of imprinting and frequent gain of chromosome 12p [14]. Despite the common embryologic origin, although potentially different in the maturation stage of the cell affected by TER and MGCT, clinical research is primarily focused on MGCT. Thus, most epidemiological cancer registries and clinical studies primarily register cases of MGCT, while TER are collected incompletely or excluded due to their benign histology. In the previous protocols (MAHO 82/88/92 and MAKEI 83/86/89), the w&w trategy had been extended to more than 50% of patients with localized MGCT [15]. Thus, the natural course of the disease could be evaluated postoperatively in such patients. 

Our study includes the largest data collection to date of children and adolescents suffering from TER as well as MGCT prospectively registered into the MAKEI 96 protocol over a 20-year period, which was approved by the responsible legal and ethical authorities. The MAKEI 96 protocol aimed to stratify the treatment of pediatric gonadal GCT according to age, localization, and stage. Backbone for the treatment of malignant pediatric gonadal GCT was in the case of locoregional or disseminated disease a cisplatin-based treatment regimen. Details regarding the stratification and dosing of chemotherapy have been published earlier [15].

The here reported data of MAKEI 96 may be informative to define the distinct epidemiological and clinical pattern of pediatric gonadal GCT according to sex, age, and histology, in a continuum of a single prospective protocol with unchanged therapeutic strategy. 

## 2. Results

### 2.1. Source and Number of Patients with TER or MGCT and Additional Findings

Between January 1996 and March 2016, 1465 patients aged 0 to <18 years with a newly diagnosed with ovarian or testicular GCT (respectively OGCT and TGCT) were registered prospectively by 62 pediatric oncology institutions from Germany, Austria, and Switzerland, of which about 90% covered about 90% of all patients with gonadal GCT aged 0 to <18 years [5,6,15]. The average annual registration rate for OGCT was 55, and for TGCT, it was 33. The majority of OGCT (630/1047, 60.2%) were TER, while the majority of TGCT (312/418, 74.6%) were malignant. Thus, incidence of TER was six-fold higher in OGCT compared to TGCT (630/1047 versus 106/418, *p* < 0.001). Since early 2000, the number of adolescents registered into MAKEI 96 with TGCT stage IIIB and IIIC (Table 3) has significantly increased, whereas only one IIIC patient had been reported before 2003 [16].

Synchronous bilateral GCT with and without metastases has been diagnosed in 60/1047 (5.7%) females (TER: 22/630 (3.5%); MGCT: 38/417 (9.1%)) but in none of the 418 male patients. Metachronous bilateral GCT has been registered in 18/1047 (1.7%) females (TER: 15/630 (2.4%), MGCT: 3/417 (0.7%)), and in 4/418 (1.0%) males (all MGCT), respectively. 

Other diseases such as genetic aberrations (e.g., 46 XY complete gonadal dysgenesis, Turner syndrome) or GCT with additional other histologies (e.g., gonadoblastoma, soft tissue sarcoma) at diagnosis were identified in 94/1465 (6.4%) patients. These 94 patients presented with 52 different diagnoses in addition to the GCT. However, the spectrum of diagnoses was completely different in boys and girls. Among phenotypically females, Swyer syndrome (i.e., XY gonadal dysgenesis) was most frequently observed (9/1047). In boys, urogenital malformations such as hypospadias and cryptorchism (9/418 pts) were most common. Among MOGCT patients, 58/417 (13.9%) had additional disorders, compared to 16/312 (5.1%) MTGCT patients (*p* < 0.001). 

#### Histology and Stage Editors

Patients with TER (n = 736) and 240/415 stage I MGCT were observed according to protocol MAKEI 96. Adjuvant therapy was applied in 175/415 stage I patients and 314 stage II–IV patients. Patients with progression after w&w strategy received chemotherapy according to the tumor stage at progression.

### 2.2. Follow-Up of Events

Tumor progression or relapse after primary tumor resection occurred in 13/630 (2.1%) OTER. These patients were cured with second resection only. Of note, 5/13 patients with the progression of an OTER died from other diseases occurring 16 months or even later after the resection of the ovarian tumor: two patients developed malignant transformation of the TER, one patient died from a preexisting medulloblastoma, one death was caused by a chronic autoimmune hepatitis, and one was caused by meningitis. None of the 106 patients with a TTER progressed, relapsed, or died. 

Lethal outcome among MGCT was reported in 10/417 (2.4%) MOGCT and 10/312 (3.2%) MTGCT patients. Patients who died had predominantly advanced disease at diagnosis (17/20 pts, 85%) and could not be cured with chemotherapy, including salvage therapy (Table 1). Comparing MOGCT and MTGCT, more events were observed among stage I MOGCT ± chemotherapy than in stage I MTGCT ± chemotherapy (MOGCT: 59/245 events (24.1%) versus MTGCT: 15/170 events (8.8%), *p* < 0.001). In contrast, the rate of events was similar for MOGCT of high stages compared to MTGCT (FIGO II-IV: 19/172 events (11.0%) versus Lugano II-IIIc 22/142 events (15.5%), *p* = 0.244).

To analyze the influence of age, patients were divided into six age groups (0 < 3; 3 < 6; 6 < 9; 9 < 12; 12 < 15; and 15 < 18 years) at first diagnosis. Table 2 illustrates the overall favorable prognosis of gonadal MGCT, in particular for prepubertal patients. All fatal outcomes were observed in patients older than 9 years: the youngest girl died at the age of 9 years, whereas the youngest boy was 12 years.

Among the 20 lethal events, three were non-tumor related (3/20 pts, 15%). One girl with preexisting RETT syndrome received no chemotherapy on parental request. Two boys died of infection after intensified chemotherapy and repeated surgery. In addition, two females were lost to follow up with active disease: a 16-year-old FIGO IB female with growing TER syndrome, and a 17-year-old female with FIGO IA-MOGCT followed by w&w who developed contralateral disease with multiple distant metastases one month after diagnosis of MOGCT. Please define if appropriate

### 2.3. Histologic Subentities

Detailed information on the histopathological diagnoses of OGCT and TGCT are provided in Table 3. Across the observed age period, the histological spectrum significantly varies with sex/site. For example, mature OTER (grade 0) are more frequent than TTER (544/630 pts (86.3%) versus 81/106 pts (76.4%), *p* < 0.001), whereas the portions of immature TER grade 1 achieves no statistically significant difference (60/640 OTER pts (9.5%) versus 14/106 TTER pts (13.2%), *p* = 0.06). Among MGCT, the histologic subentities of YST+TER and DYS dominated in MOGCT, whereas pure YST and MMGCT are the most frequent in MTGCT.

### 2.4. Occurrence of Gonadal TER and MGCT with Respect to Sex, Age, and Histology

**Figure 1:**
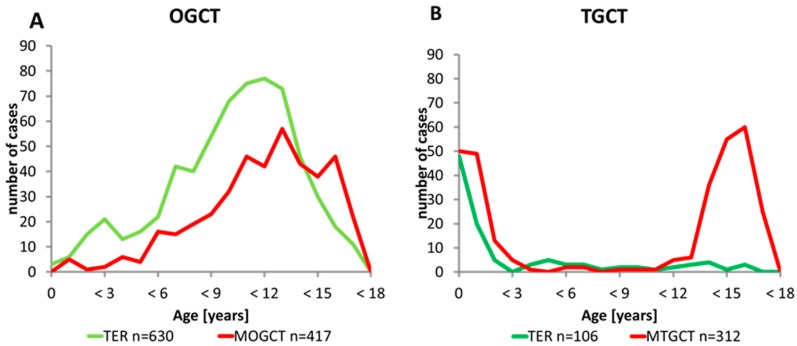
Occurrence of gonadal teratoma and malignant germ cell tumor with respect to sex and age. (**A**) Age distribution of 1047 patients with an ovarian germ cell tumor (OGCT) according to the histology groups: the green line represents the 630 teratomas (TER) and the red line represents the 417 malignant ovarian germ cell tumors (MOGCT) OGCT showed a continuously increasing incidence with a peak at approximately 12 years; (**B**) Age distribution of 418 patients with a testicular germ cell tumor (TGCT) according to the histology groups: the green line represents the 106 teratomas (TER) and the red line represents the 312 malignant testicular germ cell tumors (MTGCT). TGCT showed a bimodal age distribution.

**Figure 2:**
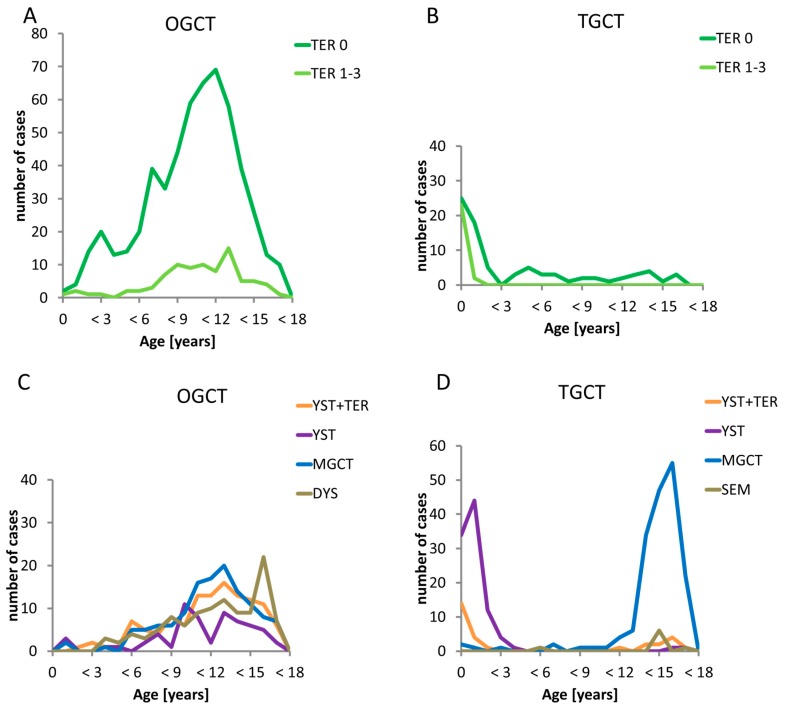
Occurrence of gonadal histology subentities with respect to sex and age. The green lines represent the benign teratomas (TER) according the grade of Gonzales-Crussi divided into grade 1 to 3 (light green) teratoma and teratoma grade 0 (dark green line). The other coloured lines represent the several malignant histological subentities: the yellow line are yolk sac tumor within teratoma components (YST+TER), the violet line represent pure yolk sac tumor, the blue line mixed malignant germ cell tumors (MGCT) and the green line pure dysgerminoma (DYS)/seminoma (SEM). (**A**) Timely occurrence of the benign subentities in ovarian germ cell tumors (OGCT). The occurrence of both benign subentites in the age groups are similar but teratomas grade 0 are occurring more often; (**B**) Timely occurrence of the benign subentities in testicular germ cell tumors (TGCT) The first peak of both teratoma grade 0 and teratomas grade 1-3 appears from birth to 3 years, whereas only teratomas grade 1-3 are seen in the other age groups; (**C**) Timely occurrence of the malignant subentities in ovarian germ cell tumors (OGCT) All malignant subentities occurred during infancy and early childhood with predominant YST histology and presented a slower rise starting at 6 years of age with a peak during adolescence for all malignant GCT subentities; (**D**) Timely occurrence of the malignant subentities in testicular germ cell tumors (TGCT). The first peak consisting of YST occurred from birth to 3 years, whereas the second peak exclusively including MTGCT started at 12 years and reached its highest point at 15 to <18 years. Between the two peaks, there is an almost complete lack of all malignant subentities over a period of 9 years

The age distribution of OGCT and TGCT is different (Figure 1A,B and Figure 2A–D). OTER showed a continuously increasing incidence with a peak at approximately 12 years (Figure 1A and Figure 2A). In comparison, MOGCT rarely occurred during infancy and early childhood with predominant YST histology and presented a slower rise starting at 6 years with a peak during adolescence and featuring all GCT subentities (Figure 1A and Figure 2C). In contrast, TGCT showed a bimodal age distribution (Figure 1B). The first peak consisting of TER and YST occurred from birth to 3 years, whereas the second peak exclusively including MTGCT started at 12 years and reached its highest point at 15 to <18 years (Figure 2B,D). Between the two peaks, there is an almost complete lack of TTER and MTGCT over a period of 9 years. 

### 2.5. Stage of Disease in Respect to Age

All ovarian teratoma (OTER) were stage I except in five females who had metastatic disease (affecting lymph nodes) or lung originating from a regionally infiltrating bilateral disease. All of them had no AFP elevation. Only one testicular TER had stage IIA due to locoregional infiltration. The primary tumor and metastatic lesions of these six patients have been resected. One patient with FIGO IIb relapsed locally after 35 months and survived in second remission after additional surgery. 

The stage distribution of MGCT (listed in respect to sex and age) shows a higher proportion of advanced disease (Lugano IIIC) in adolescent TGCT compared to OGCT (FIGO IV: 18/417 (4.3%) versus Lugano IIIC: 31/312 (9.9%); *p* < 0.001).

### 2.6. Events in w&w Patients in Respect to Age and Histology

**Figure 3:**
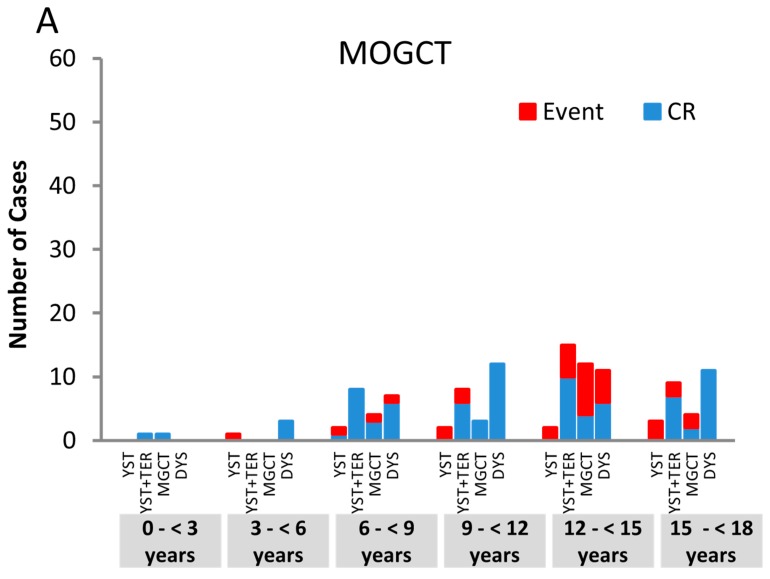

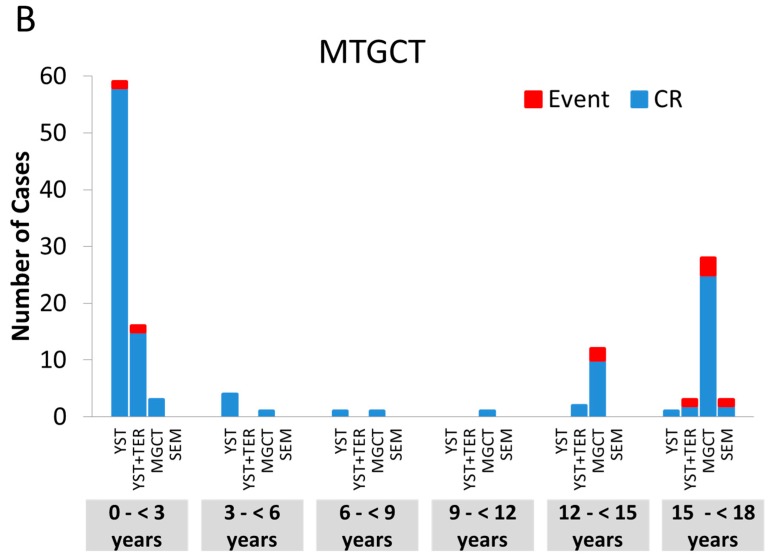
Outcome in stage I patients undergoing w&w strategy, Events (red column) are presented in respect to age and the histologic subentities. (**A**) Events in 137 FIGO stage I patients undergoing w&w strategy with malignant ovarian germ cell tumors (MOGCT): 46 Events are presented in respect to age and the histologic subentities. In MOGCT, higher age correlates with a higher progression rate, this difference is significant. Furthermore pure YST histology was associated with a high risk of progression; (**B**) Events in 103 Lugano stage I patients undergoing w&w strategy with malignant testicular germ cell tumors (MTGCT): 12 Events are presented in respect to age and the histologic subentities. In MTGCT, no age and histology specific trends were observed.

The outcome of MGCT assigned to the w&w strategy with a follow up of at least 24 months is shown in Figure 3A,B. Outcome was analyzed in respect to age and histological subgroups. In MOGCT, more events were reported compared to MTGCT (48/137 (35.0%) versus 12/103 (11.7%); *p* < 0.001).

In MOGCT, higher age correlates with a higher progression rate. Every second girl aged 12 to ≤15 years (20/40, 50%) progressed, whereas only 4/25 girls (16%) aged 9 to ≤12 years did (*p* < 0.01). In MTGCT, no age specific trend was observed. 

For MOGCT but not MTGCT, pure YST histology was associated with a high risk of progression. Nine of 10 females (9/10, 90%) with pure YST assigned to the w&w strategy progressed. In contrast, only one progression was seen among 65 pure testicular YST (1/65, 1.5%) patients, who almost were exclusively observed during infancy. 

## 3. Discussion

Pediatric gonadal and extragonadal GCT can be divided into distinct groups based on sex and age. To our knowledge, our analysis represents the largest cohort of prospectively collected pediatric patients with gonadal GCT up to date. We demonstrate distinct clinical patterns that most likely reflect biological differences. Therefore, the clinical patterns of pediatric GCT have to be discussed in the light of pre- and postnatal germ cell and gonadal development. 

The high proportion of extragonadal GCT (EGGCT) (60%) in children points to the migration routes of the PGCs from the yolk sac, where they are recognized from three to four weeks post conception (wpc), along the midline of the body, to the genital ridge. From the 5½ week of development, they are present in the gonadal location as well as in the hind gut and the mesentery, respectively [17]. Systemic anatomical research on 16 human specimens, obtained from legal abortions at 5–14 wpc, demonstrated that the PGC follow the nerve fibers of the enteric and the sympathetic nervous systems toward the gonadal site. PGC that are not leaving the nerve branches at the gonadal site, and are not eliminated by apoptosis, continue migration along the sympathetic trunk ending up in other organs; numerous PGC are still present in the nervous system by 14 wpc [18,19]. Primary sites of EGGCT have been described within the central nervous system (20%), the sacrococcygeal region (20%), the mediastinum (10%), and the CNS (20%) [1,4,17,18]. The remaining 50% are dispensed on other midline structures. 

Irrespective of tumor site, the biological type of GCT varies by age: Among gonadal GCTs, those developing in children (0–3 years) of both sexes are grouped as type I GCTs. However, MOGCTs developing in young girls (<9 years) show a genetic profile of type II GCTs, whereas in the rare TGCT of boys aged 3–12 years, only one out of 10 was identified as type II GCT [20]. These findings indicate that the presentation of gonadal and extragonadal GCT in infants, children, and adolescents may reflect different developmental pathways, which still have to be elucidated [21,22,23].

### 3.1. Genomic Imprinting Decrypts Initiation of GCT

GCT are thought to derive from PGCs or early gonocytes and their genomic imprinting pattern reflects the developmental status of pre- or postmeiotic gametes [24]. Of note, the physiological development of germ cells differs with sex. In the ovaries, PGCs proliferate during embryonal and fetal development, and thus, the number of ova is determined early in life, i.e., before birth. Prenatally, female germ cells undergo the first steps of meiosis, which are completed briefly before fertilization. In contrast, male germ cells show mitotic proliferation prenatally, and with the onset of puberty, spermatogonia continue to proliferate and undergo meiosis throughout life. Thus, physiologically millions of sperm cells develop per day. Imprinting analyses have demonstrated that tumor initiation of gonadal and non-gonadal GCT appears to fall into the early phases of PGC development, as in most type I GCT, an immature erased imprinting pattern is preserved, which is erased in type II [24]. Only, ovarian TER may show an isodisomic karyotype, with a female genomic imprinting pattern. Both findings indicate that these tumors, in contrast to TER at other sites, develop from postmeiotic female germ cells [12,25]. 

### 3.2. Proliferation of GCT

While the clinical occurrence of GCT at different sites is the result of migration and proliferation of germ cells beside the gonads, it may fall into different age periods, and sex-specific patterns can be observed. Type I GCT only occur in infants and young children, with rare exceptions [26,27]. Their histological pattern is restricted to TER and YST, and they show characteristic chromosomal patterns such as loss of chromosome 6q and imbalances of chromosome 1. In contrast, the vast majority of type II GCT develop after the onset of puberty and throughout adulthood. These may present with the complete histological spectrum and are characterized by a gain of chromosome 12p, e.g., isochromosome 12p [28]. Therefore, this clinical observation demonstrates that both normal and initiated/transformed PGC may survive over decades but may then proliferate after a yet undiscovered specific trigger, either in a physiological or pathological pattern. 

Of note, TGCT may present as both type I or II GCT with the above-mentioned distinct histological and genetic patterns. In our cohort, type I TGCT occurring in early childhood contribute about 50% of all observed TGCT until an age of 18 years. In contrast, less than 3% of OGCT were diagnosed below the age of 3 years (Figure 1A,B). In particular, for DYS and seminoma (SEM), the incidence peak lies after the age of 18 years. This may indicate a distinctive role of the sex chromosomal status and the underlying intrinsic developmental pattern. In males, testicular dysgenesis syndrome includes a spectrum of developmental perturbances such as hypospadias, cryptorchism, and poor semen quality, and it is associated with an increased risk of TGCT, which relates to the risk of postpubertal type II TGCT, only [29]. In females, OGCT may develop in the context of sex chromosomal disorders (e.g., Swyer syndrome) [30,31]. Patients with different forms of gonadal dysgenesis are at an increased risk of developing gonadoblastoma, being the precursor lesion of dysgenetic gonads, such as Germ Cell Neoplasia in Situ of the testis (GCNIS) and subsequent type II GCT [32].

### 3.3. Teratoma (TER) and Malignant Germ Cell Tumors (MGCT)

One of the most important aspects of the MAKEI dataset was the ability to collect prospective data on TER. In our series, testicular mature and immature TER represent a first step in oncogenesis Figure 2B,D) characterized by uncoordinated tumor growth and a loss of induction of apoptosis [33], as also described in evaluations of parts of our cohort [34]. In contrast, the rare testicular pure TER of adolescents is often malignant and may show a gain of chromosome 12 comparable to typical type II tumors. The age at presentation of ovarian TER of our sample precedes that of MOGCT (Figure 2A,C). However, it can be postulated that OTER do not biologically precede malignant OGCT, as their biology is completely different [35]. As described by Bussey et al. 1999, ovarian TER are isodisomic and develop from postmeiotic germ cells, while MOGCT show diploid or aneuploid karyotype with specific aberrations of type II GCT such as 12p gain. Nevertheless, about 3% of ovarian mature TER developed single metastases with a favorable prognosis, whereas the immature TER of single adolescents rarely presented with poor prognostic widespread disease [36].

Out of our cohort with ovarian TER (n = 630), five patients died (0.8%), but none among the 106 patients with a testicular TER died. As death of these five patients was not connected to the TER, they have to be considered a coincidence. Otherwise, malignant transformed metastatic TER or growing teratoma syndrome portend a poor prognosis. However, these are only rare events in patients <18 years [37]. 

Our analysis also clearly outlines the interval between early childhood and the onset of puberty, with an extremely low incidence of MGCT. In the MAKEI 96 cohort, the very few MOGCT in girls < 6 years (n = 18) also show a defined histologic pattern (Figure 3C) similar to type II GCT. The few MTGCT in boys aged 3 to 12 years (n = 13) may show overlapping features of type I or type II GCT (Figure 2D) [38]. This phenomenon has been reported before [35].

The clinical presentation of MGCT in MAKEI 96 also changes with the onset of puberty and shows sex-specific patterns. After the onset of puberty, there is a shift from non-metastatic stage I MTGCT (90% in children <12 years) to metastatic stage II and III (30% and 39%, respectively, in adolescents >12 years) tumors. In contrast, the majority of OGCT (59%) present with FIGO stage I even despite the considerable size of many tumors, while tumors with regional spread (FIGO II/III, 37%) or distant metastases (FIGO IV, 4%) remain rare events. Therefore, gender-specific differences may also affect the clinical presentation even in the subgroup of type II GCT.

Interestingly, synchronous bilateral GCT were not observed in males, and metachronous bilateral GCT did also appear mainly in girls. These observations have not been reported elsewhere to our knowledge. A further discussion of these results will be the subject of a separate publication.

### 3.4. Biologic Heterogeneity Despite Comparable Morphology

These clinical patterns may potentially correlate with different triggers or altered signaling cascades. There are many clinical findings that point to biological differences that may affect signal cascades involved in the regulation of proliferation in resting initiated/transformed PGC and of their malignant transformation. This includes the two incidence peaks of TGCT, the almost complete absence of MOGCT during the first six years of life, and the occurrence of metachronous bilateral gonadal GCT both in the testes and ovaries, as shown in our evaluation. 

Therefore, despite the comparable morphological appearance within the histological subentities over age, the group of gonadal GCT is highly heterogeneous. Specific subgroups have to be defined, and the prognostic impact of the different classification systems in respect to tumor cell dissemination should be reevaluated according to age [39,40]. Among the w&w patients of our cohort (Figure 3A,B) there is a trend (*p* = 0.058) to a higher risk of progression according to age. Compared to TGCT, significantly more OGCT progressed. However, we describe a significant increase of Lugano stage III TGCT in adolescents. These trends may potentially correlate with the accelerated proliferation of male germ cells after puberty and the decreased spontaneous apoptosis of transformed PGC. 

### 3.5. Patients with Best and Worst Prognosis

Apparently, the low metastatic potential and the high ability to undergo platinum-induced apoptosis are the keys for the excellent prognosis of young boys with type I GCT. Only 23% of type I TGCT required adjuvant chemotherapy, and all were cured [41]. The proportion of type II TGCT aged 10 to 15 years requiring adjuvant chemotherapy for advanced stage was higher, but cure rates were still excellent [16]. However, this cohort of patients contained only one single patient with LUGANO stage IIIC. Due to changes in German legal regulations, since 2006, adolescents up to the age of 18 years have to be treated in children’s hospitals. This led to an increase specifically in the number of registered male adolescents aged 15 to 18 years. Stage III was observed in every third of these patients, and the worst cases with TOGCT were predominantly found in this age group. In OGCT, the characteristics of worst cases are distributed differently: In our series, 9/417 (2.2%) females aged 9 to 16 years died as a result of tumor progression compared to 8/312 (3.2%) males, all of whom were of pubertal age. Most patients presented with far advanced diseases at diagnosis, but two girls had bilateral tumors with malignant transformation (Table 2). The other female patients, who developed early progression under treatment and died, can be considered to have a primary cisplatin resistance. These all constitute rare events. However, they may point out a different risk profile of testicular and ovarian GCT. 

## 4. Methods

### 4.1. Protocol

From the mid 1980s onwards, two pediatric GCT protocols have existed for testicular (MAHO, approved by the Ethics Committees of the Ludwig-Maximilian-University München), and for non-testicular (MAKEI, approved by the Ethics Committee of the Heinrich-Heine-University Düsseldorf) GCT in Germany. In 2003, both protocols were merged to one protocol, MAKEI 96, which has been approved by the Ethics Committee of the Heinrich-Heine-University Düsseldorf and the protocol review committee of the German Cancer Society. The patients or their legal representative provided written consent for data registration and transfer after pseudonymization as well as treatment according to the protocol. Patients without written consent, age ≥18 years, tumors without GCT histology, missing important information, or first registration at relapse were excluded.

### 4.2. Diagnosis and Staging

Diagnosis was based on conventional histology and immunohistochemistry by the local pathologist and assessment of serum tumor markers (Alpha feto protein (AFP) and Beta-human chorionic gonadotropin (ß-HCG)). For the vast majority of patients, a central pathological review was performed at the German Kiel Pediatric Tumor Registry for the verification of diagnosis and classification according to the revised WHO classification for TGCT [42,43,44].

Assignment to the different histological GCT entities was made according to the following principles: Tumors containing at least two malignant histologies (i.e., yolk sac tumor (YST), embryonal carcinoma choriocarcinoma, and/or seminoma (testis)/dysgerminoma (ovary) (GER)) were termed mixed malignant GCT (MMGCT). The rare pure embryonal carcinoma or choriocarcinom were also assigned to the MMGCT. The combination of YST+TER, which was regularly observed in infants, represents its own subentity. If abnormal elevated serum AFP or ß-HCG were detected, tumors were considered as MGCT even if malignant histology was not detectable in the analyzed tumor specimen. Mature (grade 0) and immature TER (grade 1–3) were categorized according to Gonzales-Crussi. Bilateral GCT patients with different histologies were stratified according to the most malignant component. 

Clinical and radiological staging included pelvic and abdominal ultrasound, MRI, or alternatively CT, chest X-ray, brain MRI (in case of neurological symptoms), routine blood chemistry, and measurement of the tumor markers AFP and ß-HCG. 

### 4.3. Tumor Resection

For OGCT, the surgical approach, laparotomy versus laparoscopy, depended on the level of preoperative suspicion of malignancy, as laparotomy reduces the risk of spillage and was mandatory in case of malignant tumor [45]. For anticipated MOGCT, tumor resection together with the ovary was recommended via an open surgical approach, and salpingo-oophorectomy was recommended in case of infiltration of the adnexa or the small pelvis. In case of suspected TER, ovarian-sparing surgery could be performed either open or laparoscopically [46]. TGCT were resected via a high inguinal approach. OGCT were staged according to the FIGO classification [39] and TGCT according to the LUGANO classification [40]. Additional findings, such as second malignancies, associated genetic syndromes, or urogenital malformations, were documented. 

### 4.4. Statistical Evaluation

Data have been continuously monitored at the central statistical center at the University Hospital of Bonn, where the database has been hosted since 2016. 

Treatment response was assessed according to the Response Evaluation Criteria in Solid Tumors guidelines (RECIST) version 1.0. Tumor progression after the w&w strategy, locoregional or metastatic relapse, and death from any cause were recorded as events. Event-free survival (EFS) was defined as the interval between diagnosis and the time of last follow-up visit. Overall survival (OS) was defined as the interval between diagnosis and the time of last follow-up visit or death. Patients who achieved a second remission after relapse (REL), who died due to tumor progression (DOD), or died due to other reasons (DOR) were analyzed separately. SAS (Version 9.2 for Windows; SAS Institute Cary, NC) was used for statistical analysis. Microsoft Excel was used for table calculations and illustration of diagrams.

To analyze the influence of age, patients were divided into six age groups (0 < 3; 3 < 6; 6 < 9; 9 < 12; 12 < 15; and 15 < 18 years at diagnosis). The complete cohort was analyzed according to a risk-adapted hierarchical decision tree model including the parameters sex/site, TER versus MGCT, age at first diagnosis, tumor stage, and treatment strategy [47].

To evaluate potential differences between the various examined groups, the following statistical tests were applied: Categorical parameters were compared using the χ^2^-Test of Independence. Numeric parameters were compared using non-parametric tests for two (Wilcoxon–Mann–Whitney Test) or more groups (Kruskal–Wallis Test) with the common significant barrier of 5%.

## 5. Conclusions

Our evaluation clearly points out that age is the most important prognostic risk factor in pediatric GCT. TER are the most common GCT in childhood. Our data, representing the largest series of TER to date, show a distinct difference in behavior between OTER and TTER, which underlines the influence of sex in the development and presentation of this disease. Testicular mature/immature TER in the young do not show chromosomal aberrations and may represent a first step in oncogenesis characterized by uncoordinated tumor growth and loss of induction of apoptosis. In contrast, the rare testicular pure TER of adolescents are often malignant and may show a gain of chromosome 12 comparable to typical type II tumors. 

The observed overall survival data of MGCT in our series are comparable to those reported in other actual protocols. As the duration of treatment was consecutively reduced for high-risk patients since the early 1980s, and as the group of w&w patients enlarged concommitantly, the importance of an elaborate diagnostic work-up and the need for expert knowledge for treatment of such patients have become increasingly clear. 

Platinum-induced apoptosis may overcome most risk factors of MGCT in patients aged 0 to 18 years, but some of the patients progress during or after standard therapy, and most of these may be cured with additional treatment, such as repeated surgeries, irradiation [48], and/or additional hyperthermia [49]. 

Other patients die despite early intensive platinum-based combination chemotherapy. These point to the complexity of platin sensitivity and resistance that may result from multiple epigenetic and genetic changes [50]. This relates in particular to adolescent MGCTs who presented with advanced stages and progressed early during treatment. This phenomenon was observed in MTGCT and MOGCT with a slight difference in frequency, as in MOGCT, other causes of death such as malignant transformation were described.

Therefore, research should be directed to identify the signaling cascades relevant in well-defined risk groups.

The rarity of such and other events impairs the so-far excellent prognosis of pediatric GCT and urges for more international cooperation [51]. This analysis may be helpful as a baseline for further investigation and may be relevant for the future stratification of therapy. As older male adolescents are higher at risk, the stratification has to be done in accordance with the European guidelines for TGCT [52].

## Figures and Tables

**Table 1:** Events in patients with localized and advanced malignant germ cell tumors (MGCT) in respect to stage and treatment strategy. MOGCT: malignant ovarian germ cell tumors, MTGCT: malignant testicular germ cell tumors.

Tumor Site	Treatment Strategy	Patients	Progression/Relapse and Alive	Died
MOGCT	FIGO I w&w	137	46 (33.6%)	2 (1.5%)
	FIGO I + chemo	108	11 (10.2%)	0 (0%)
	FIGO II−IV + chemo	172	11 (6.4%)	8 (4.7%)
MTGCT	Lugano I A/C w&w	103	12 (11.7%)	0 (0 )
	Lugano I A/C + chemo	67	2 (3.0%)	1 (1.5%)
	Lugano II−IIIC + chemo	142	13 (9.2%)	9(6.3%)

**Table 2:** Patients (pts) with MGCT dead of disease (DOD), lethal infection (DOI), or other reason (DOR) in respect to the six age groups.

Tumor Site	Age	0 to <3	3 to <6	6 to <9	9 to <12	12 to <15	15 to <18
MOGCT	all pts	6	12	50	101	142	106
	DOD	-	-	-	4	3 ¹	2 ¹
	DOR	-	-	-	-	1 ^2^	-
MTGCT	all pts	112	6	4	3	47	140
	DOD	-	-	-	-	3 ^3^	5
	DOI	-	-	-	-	-	2

¹ One pt each offered malignant transformation (glioblastoma multiforme, squamous cell carcinoma; ^2^ Chemotherapy was refused because of RETT syndrome (RTT) (see abbreviations); ^3^ One pt with central nervous system (CNS) metastases died with signs of elevated cerebral pressure on day 9 after the start of chemotherapy.

**Table 3:** Histologic subentities of the patients aged 0 to <18 years. TER: teratomas, YST: yolk sac tumor.

Histology	OGCT n (*%*)		TGCT n (*%*)		*p*
TER Σ	630		106		
mature TER (grade 0)	544	(86.3)	81	(76.4)	<0.001
immature TER grade 1	60	(9.5)	14	(13.2)	0.24
immature TER grade 2	22	(3.5)	8	(7.5)	0.05
immature TER grade 3	4	(0.6)	3	(2.8)	0.03
MGCT Σ	417		312		
YST + TER	119	(28.5)	29	(9.3)	<0.001
pure YST	62	(9.2)	98	(31.4)	<0.001
pure EC	1	(0.2)	9	(2.9)	<0.002
pure CC	11	(2.6)	5	(1.6)	0.360
MMGCT	115	(27.6)	163	(52.2)	<0.001
DYS respectively SEM	109	(26.1)	8	(2.6)	<0.001
TER + MGCT Σ	1047		418		

## Abbreviations

AFP	Alpha feto protein
ß-HCG	Beta-human chorionic gonadotropin
CHC	Choriocarcinomas
CNS	Central Nervous System
CR	Complete Remission
CT	Computer tomography
DOD	Dead of Disease
DOI	Dead of Lethal Infection
DOR	Dead of Other Reason
DYS	Dysgerminomas
GCT	Germ Cell Tumors
GCNIS	Germ Cell Neoplasia in situ of the Testis
EFS	Event Free Survival
EGGCT	Extragonadal Germ Cell Tumors
GER	Germinomas
IGCCC	International Germ Cell Classification Consensus
MEGCT	Malignant extragonadal Germ Cell Tumors
MGCT	Malignant Gonadal Germ Cell Tumors
MMGCT	Mixed malignant Germ Cell Tumors
MOGCT	Malignant ovarian Germ Cell Tumors
MRI	Magnet-resonance-imaging
MTGCT	Malignant testicular Germ Cell Tumors
OGCT	Ovarian Germ Cell Tumors
OS	Overall Survival
pts	Patients
OTER	Ovarian Teratoma
PGC	Primordial Germ Cells
RECIST	Response Evaluation Criteria in solid Tumors Guidelines
RTT	Rett syndrome is a genetic brain disorder, apparent after 6 to 18 months of age in females. Symptoms include problems with language, coordination, and repetitive movements, additionally slower growth, walking problems, and a smaller head size.
REL	Relapse
SEM	Seminomas
TER	Teratomas
TTER	Testicular Teratomas
TGCT	Testicular Germ Cell Tumors
w&w	Watchful Waiting
wpc	Weeks Post conception
YST	Yolk Sac Tumors
